# Dental Arch Dimensions in Saudi Adults

**DOI:** 10.1155/2018/2190250

**Published:** 2018-02-15

**Authors:** Omar Hamad Alkadhi, Sarah Fahad Almahfouz, Hana Abdulmajeed Tokhtah, Lamia Abdulaziz Binhuwaishel

**Affiliations:** ^1^Division of Orthodontics, Department of Preventive Dentistry, Riyadh Colleges of Dentistry and Pharmacy, Riyadh, Saudi Arabia; ^2^Saudi Board of Orthodontics, Department of Orthodontics, King Saud University, Riyadh, Saudi Arabia; ^3^Orthodontics Residency Program, King Saud University, Riyadh, Saudi Arabia; ^4^Department of Orthodontics, King Saud Medical City, Riyadh, Saudi Arabia

## Abstract

**Objective:**

The aim of this study was to investigate the arch dimensions (width, length, and depth) in Saudi Arabia.

**Materials and Methods:**

169 orthodontic models (73 males and 96 females) made of white stone were selected according to inclusion criteria. Measurement of arch dimensions was taken including width, length, and depth at different reference points. Differences between males and females were tested using independent samples *t*-test.

**Results:**

The sample comprised subjects aged 18–33 years old. Most measurements showed higher values for males compared to females. Saudi males showed smaller intercanine widths compared to Caucasians and Southern Chinese but larger widths in females compared to Caucasians and smaller widths compared to Southern Chinese. Saudi males and females showed larger intermolar widths compared to Caucasians but smaller intermolar widths compared to Southern Chinese. For arch length, Saudis showed longer arches compared to Yemenis for both males and females but smaller palatal depths compared to Yemenis.

**Conclusions:**

Differences in intercanine width, intermolar width, and palatal length and depth were found between Saudis and other nationalities. Male and female participants had differences in most of the measurements.

## 1. Introduction

Dental arch dimensions are of special interest for dentists and orthodontists in particular. Changes in the arch width, length, and height can result from orthodontic treatment; hence, an understanding of the dental arch dimensions is crucial [1–5]. Dental arches have been investigated using different measurements and reference points, including but not limited to, intercanine, interpremolar, and intermolar widths, either between cusps or fossae, anterior palatal and mandibular lengths, molar vertical distance, total palatal and mandibular lengths, and palatal depth [6–11].

Ward et al. have reported significant changes in maxillary and mandibular intercanine widths in orthodontically treated group compared to untreated group [12]. In patients treated with Frankel II appliance, significant increases in arch width and arch perimeter were found [13].

There are many studies that have been conducted to investigate the dental arch dimensions and relationships in different ethnicities and different geographical areas [14–20]. Few studies have been conducted to investigate arch dimensions in Saudi population [21, 22]. Therefore, the aim of this study was to investigate the arch dimensions (width, length, and depth) in Saudi adults.

## 2. Materials and Methods

This study was registered at the research center at Riyadh Colleges of Dentistry and Pharmacy with registration number: FRP/2014/73 and IRB approval was obtained. Inclusion criteria were as follows:

1. Saudi subjects over 18 years of age
2. Class I canine and molar relationships
3. Presence of all permanent teeth up to the second molars
4. No previous orthodontic treatment or facial surgeries
5. No more than mild crowding or spacing (<3 mm)
6. No history of parafunctional oral habits
7. No large restorations or crowns
8. No tooth anomalies

**Figure 1:**
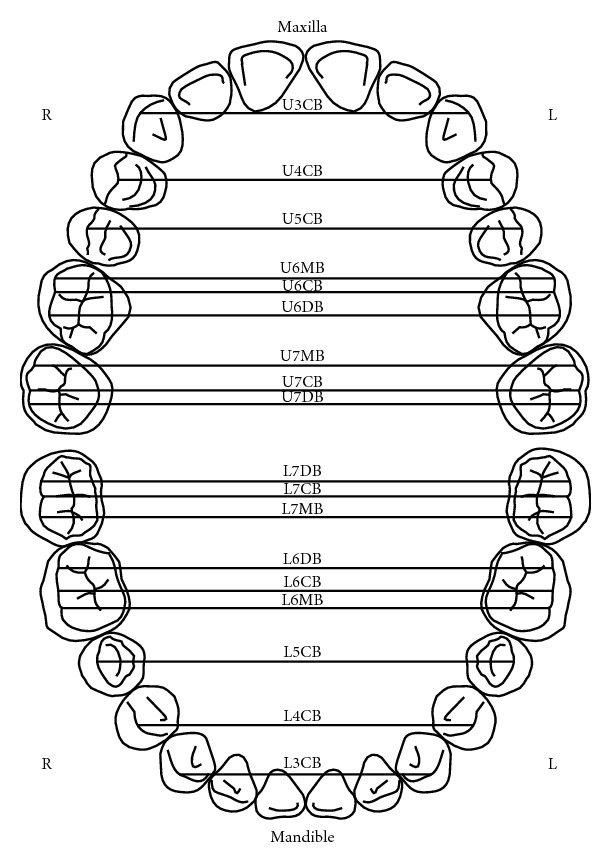
Illustration of the maxillary and mandibular arches' widths as described by Ling and Wong [9]. U3CB: cusp tip of tooth 13 and tooth 23; U4CB: buccal cusp of tooth 14 and tooth 24; U5CB: buccal cusp of tooth 15 and tooth 25; U6MB: mesial buccal cusp of tooth 16 and tooth 26; U6CB: buccal groove at the buccal and occlusal surface interface of tooth 16 and tooth 26; U6DB: distal buccal cusp of tooth 16 and tooth 26; U7MB: mesial buccal cusp of tooth 17 and tooth 27; U7CB: buccal groove at the buccal and occlusal surface interface of tooth 17 and tooth 27; U7DB: distal buccal cusp of tooth 17 and tooth 27; L7DB: distal buccal cusp of tooth 37 and tooth 47; L7CB: buccal groove at the buccal and occlusal surface interface of tooth 37 and tooth 47; L7MB: mesial buccal cusp of tooth 37 and tooth 47; L6DB: distal buccal cusp of tooth 36 and tooth 46; L6CB: buccal groove at the buccal and occlusal surface interface of tooth 36 and tooth 46; L6MB: mesial buccal cusp of tooth 36 and tooth 46; L5CB: buccal cusp of tooth 35 and tooth 45; L4CB: buccal cusp of tooth 34 and tooth 44; L3CB: cusp tip of tooth 33 and tooth 43.

**Figure 2:**
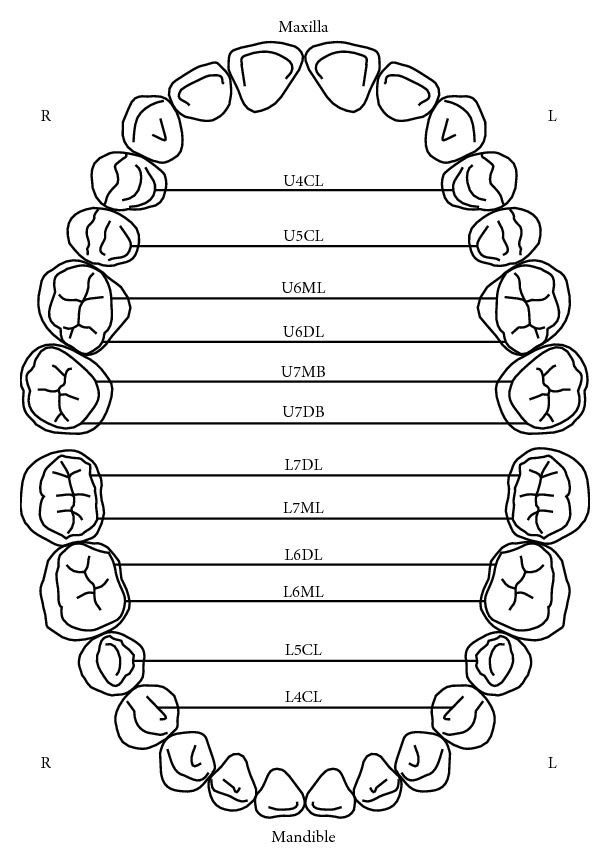
Illustration of the maxillary and mandibular arches' widths as described by Ling and Wong [9]. U4CL: palatal cusp of tooth 14 and tooth 24; U5CL: palatal cusp of tooth 15 and tooth 25; U6ML: mesial palatal cusp of tooth 16 and tooth 26; U6DL: distal palatal cusp of tooth 16 and tooth 26; U7MB: mesial buccal cusp of tooth 17 and tooth 27; U7DB: distal buccal cusp of tooth 17 and tooth 27; L7DL: distal palatal cusp of tooth 37 and tooth 47; L7ML: mesial lingual cusp of tooth 37 and tooth 47; L6DL: distal lingual cusp of tooth 36 and tooth 46; L6ML: mesial lingual cusp of tooth 36 and tooth 46; L5CL: lingual cusp of tooth 35 and tooth 45; L4CL: lingual cusp of tooth 34 and tooth 44.

**Figure 3:**
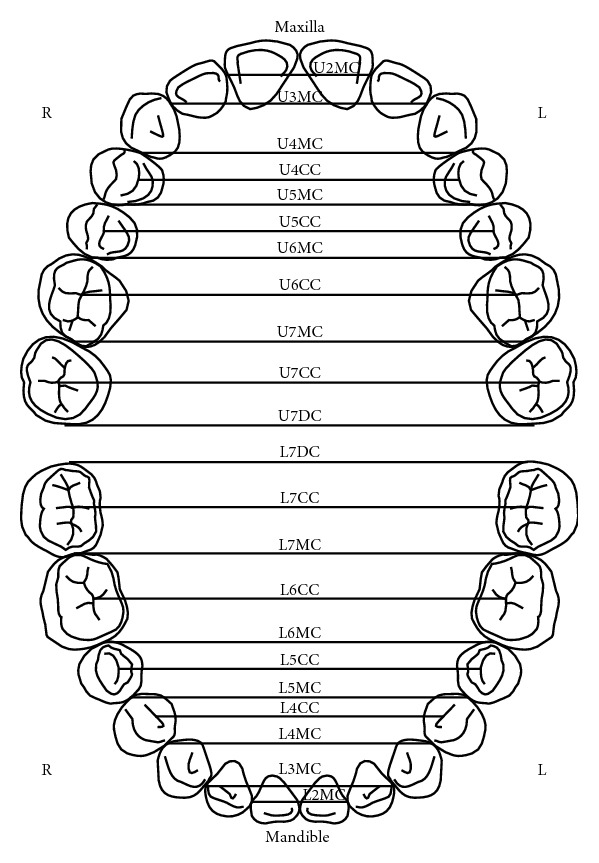
Illustration of the maxillary and mandibular arches' widths as described by Ling and Wong [9]. U2MC: contact points between tooth 11 and tooth 12 and tooth 21 and tooth 22; U3MC: contact points between tooth 12 and tooth 13 and tooth 22 and tooth 23; U4MC: contact points between tooth 13 and tooth 12 and tooth 23 and tooth 22; U4CC: central fossa of tooth 14 and tooth 24; U5MC: contact points between tooth 14 and tooth 15 and tooth 24 and tooth 25; U5CC: central fossa of tooth 15 and tooth 25; U6MC: contact points between tooth 15 and tooth 16 and tooth 25 and tooth 26; U5CC: central fossa of tooth 15 and tooth 25; U7MC: contact points between tooth 16 and tooth 17 and tooth 26 and tooth 27; U7CC: central fossa of tooth 17 and tooth 27; U7DC: estimated distal contact points between tooth 17 and tooth 18 and tooth 27 and tooth 28; L7DC: estimated distal contact points between tooth 37 and tooth 38 and tooth 47 and tooth 48; L7CC: central fossa of tooth 37 and tooth 47; L7MC: contact points between tooth 36 and tooth 37 and tooth 46 and tooth 47; L6CC: central fossa of tooth 36 and tooth 46; L6MC: contact points between tooth 35 and tooth 36 and tooth 45 and tooth 46; L5CC: central fossa of tooth 35 and tooth 45; L5MC: contact points between tooth 34 and tooth 35 and tooth 44 and tooth 45; L4CC: distal fossa of tooth 34 and tooth 44; L4MC: contact points between tooth 33 and tooth 34 and tooth 43 and tooth 44; L3MC: contact points between tooth 33 and tooth 32 and tooth 43 and tooth 44; L2MC: contact points between tooth 32 and tooth 31 and tooth 42 and tooth 41.

**Figure 4:**
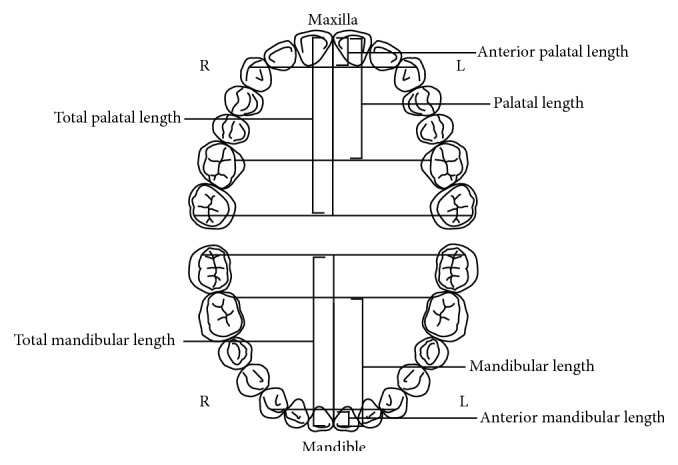
Illustration of the maxillary and mandibular arches' lengths as described by Al-Zubair [11]. Anterior palatal length: the vertical distance from the incisal point to the intercanine distance line; molar vertical distance (palatal length): the vertical distance from the incisal point perpendicular to a line between the mesial lingual cusp tips of the first molars; total palatal length: the vertical distance from the incisal point to the midpoint of a line between the distal buccal cusp tips of the second molars; anterior mandibular length: the vertical distance from the incisal point to the intercanine distance line; molar vertical distance (mandibular length): the vertical distance from the incisal point perpendicular to a line between the mesial lingual cusp tips of the first molar; total mandibular length: the vertical distance from the incisal point to the midpoint of a line between the distal buccal cusp tips of the second molar.

Orthodontic models (*n* = 169; 73 males and 96 females) made of white stone in the orthodontic clinics were examined; those that satisfied the inclusion criteria were selected. Measurements of arch dimensions of the maxilla and mandible (width, length, and depth) were taken as described by Ling and Wong [9] and Al-Zubair [11]. Definitions and illustrations of all variables are shown in Figures 1–4 and Table 1. Two examiners took the measurements using a digital sliding caliper (Carrera Precision CP9806-TF, Max Tool LLC, Calif., USA).

## 3. Method Error

For the purpose of calibration, the two examiners took all the measurements on 20 dental models and then their measurements were compared.

### 3.1. Statistical Analysis

Independent sample *t*-test was used to determine any statistically significant differences between males and females for each measurement. Interclass correlation coefficient was used to determine interexaminer reliability. SPSS software was used for the statistical analysis (IBM SPSS Statistics for Mac, Version 24.0, IBM Corp., Armonk, NY).

## 4. Results

The sample consisted of 169 individuals (male: *n* = 73, aged 23.9 years ± 4.5; female: *n* = 96, aged 23.8 years ± 4.3). All results are shown in Tables 2–5. All maxillary and mandibular measurements showed statistically significant greater values for males compared to females (*t*-test; *P* < 0.05) except for U7ML, U4CB, U3CL, U3CB, U3MC, U2MC, L5CL, L5CG, L4CC, L4CB, L2MC, APL, PL, TPL, AML, ML, and PD (*t*-test; *P* > 0.05). Interexaminer reliability was high, ranging from 0.88 to 0.92 (*P* < 0.05).

## 5. Discussion

In the present study, an effort was made to establish normal values for some parameters that have never been studied in Saudi. We chose to conduct measurements using many reference points described in the literature to allow for comparison with other populations' measurements [9, 11].

Comparison of intercanine widths among different populations showed small differences in Saudis between the present study and other studies and close measurements compared to Caucasians [20–22], while Southern Chinese showed the largest arch widths in the canine area (Table 6) [9].

Comparisons of the arch widths at the first molar showed a difference of about 1 mm between Saudis in our studies and Saudis in other studies. Southern Chinese were found to have the largest arch width followed by Saudis and then by Caucasians [9, 20] (Tables 7 and 8).

Maxillary arch length was found to be larger in Saudis compared to Yemenis in all measurements, while palatal depth slightly larger in Yemenis compared to Saudis [11] (Table 9).

The measurements established in the present study can serve as a database to which, orthodontic treatment of Saudi adults can be planned. Limitations of this study include a relatively small sample and that the study was confined to Riyadh City.

## 6. Conclusions

- This study serves the purpose of establishing a database for arch dimensions for Saudi adult population.
- Comparisons between Saudis and other populations showed differences in intercanine width, intermolar width, and palatal length and depth.
- Males and females showed statistically significant differences in most of the measurements where males had larger measured values.

## Figures and Tables

**Table 1:** Definitions of palatal depth as described by Al-Zubair [11].

Measurement	Definition
Palatal depth	The vertical distance from a point on the palatal width line to the palatal vault in the midline at the mesial palatal cups of 1st molars

**Table 2:** Upper and lower molar measurements including independent sample *t*-tests comparing males to females.

Abbreviation	Gender	*n*	Mean	Standard error of mean	Standard deviation	Minimum	Maximum	Significance (2-tailed)	Significance	Mean difference	Standard error difference	95% confidence interval of the difference	
												Lower	Upper
*Upper molars*													
U7CC	M	73	50.69	0.31	2.66	45.81	57.80	0.000	^∗∗∗^	1.70	0.42	0.88	2.53
	F	96	48.98	0.28	2.70	44.49	54.71						
U7ML	M	73	44.33	0.71	6.09	5.59	53.17	0.230	NS	1.00	0.83	−0.64	2.64
	F	96	43.33	0.48	4.72	4.87	49.44						
U7MG	M	73	39.98	0.48	4.07	20.93	50.92	0.026	^∗^	1.25	0.56	0.15	2.35
	F	96	38.73	0.32	3.16	30.76	47.38						
U7DL	M	73	46.77	0.41	3.52	40.49	58.30	0.001	^∗∗^	1.65	0.50	0.66	2.64
	F	96	45.12	0.30	2.98	40.46	52.25						
U7DC	M	73	51.23	0.43	3.69	45.50	64.89	0.000	^∗∗∗^	1.86	0.51	0.85	2.86
	F	96	49.38	0.30	2.93	45.05	56.57						
U7DB	M	73	57.98	0.32	2.75	53.12	64.31	0.001	^∗∗^	1.77	0.51	0.76	2.78
	F	96	56.22	0.37	3.65	51.07	72.79						
U7CB	M	73	57.43	0.33	2.84	52.43	64.54	0.000	^∗∗∗^	2.14	0.46	1.24	3.04
	F	96	55.29	0.31	3.01	50.26	62.33						
U7MB	M	73	56.68	0.33	2.80	51.57	63.35	0.000	^∗∗∗^	1.63	0.45	0.74	2.52
	F	96	55.05	0.30	2.98	49.46	61.95						
U7MC	M	73	49.16	0.44	3.75	43.97	58.63	0.000	^∗∗∗^	2.69	0.53	1.63	3.74
	F	96	46.47	0.32	3.18	38.26	58.10						
U6CC	F	73	45.38	0.34	2.90	39.96	52.32	0.000	^∗∗∗^	1.97	0.49	1.00	2.93
	M	96	43.42	0.34	3.32	38.25	53.58						
U6ML	M	73	39.49	0.34	2.87	33.65	46.72	0.000	^∗∗∗^	1.91	0.51	0.90	2.92
	F	96	37.58	0.36	3.57	27.81	47.94						
U6MG	M	73	33.80	0.33	2.83	23.12	40.58	0.000	^∗∗∗^	1.66	0.46	0.76	2.57
	F	96	32.14	0.31	3.05	27.54	40.58						
U6DL	M	73	41.70	0.35	2.95	36.41	49.31	0.001	^∗∗^	1.73	0.49	0.76	2.71
	F	96	39.97	0.34	3.35	32.26	53.72						
U6DB	M	73	53.25	0.33	2.80	48.97	61.95	0.000	^∗∗∗^	2.02	0.48	1.07	2.97
	F	96	51.23	0.34	3.30	41.35	58.26						
U6CB	M	73	52.10	0.31	2.61	45.87	59.11	0.000	^∗∗∗^	2.02	0.44	1.14	2.89
	F	96	50.09	0.31	3.03	42.51	56.82						
U6MB	M	73	51.03	0.36	3.08	36.44	56.78	0.000	^∗∗∗^	1.83	0.48	0.88	2.77
	F	96	49.21	0.31	3.06	44.25	56.51						
U6MC	M	73	43.21	0.32	2.75	37.66	49.38	0.000	^∗∗∗^	1.75	0.43	0.90	2.60
	F	96	41.45	0.29	2.79	35.96	47.88						
*Lower molars*													
U6MC	M	73	43.21	0.32	2.75	37.66	49.38	0.000	^∗∗∗^	1.75	0.43	0.90	2.60
	F	96	41.45	0.29	2.79	35.96	47.88						
L7CC	M	73	46.82	0.35	2.97	40.78	55.16	0.000	^∗∗∗^	2.19	0.44	1.32	3.06
	F	96	44.63	0.28	2.72	39.90	51.71						
L7DL	M	73	43.18	0.37	3.13	36.28	49.91	0.000	^∗∗∗^	2.68	0.43	1.83	3.52
	F	96	40.51	0.25	2.44	35.64	45.60						
L7DG	M	73	40.24	0.31	2.63	33.31	45.58	0.000	^∗∗∗^	2.34	0.39	1.57	3.11
	F	96	37.90	0.25	2.41	32.95	43.49						
L7ML	M	73	39.87	0.32	2.77	34.60	44.37	0.000	^∗∗∗^	1.84	0.40	1.04	2.63
	F	96	38.04	0.25	2.45	33.00	42.89						
L7MC	M	73	44.25	0.32	2.73	39.50	49.42	0.000	^∗∗∗^	2.19	0.42	1.37	3.02
	F	96	42.06	0.27	2.64	37.93	48.76						
L7CB	M	73	50.60	0.42	3.56	34.14	56.56	0.000	^∗∗∗^	2.44	0.55	1.35	3.52
	F	96	48.17	0.36	3.51	39.37	56.24						
L7DB	M	73	53.32	0.45	3.83	36.10	60.00	0.000	^∗∗∗^	2.73	0.58	1.58	3.87
	F	96	50.59	0.38	3.68	40.55	58.40						
L7DC	M	73	49.66	0.39	3.29	42.53	57.21	0.000	^∗∗∗^	2.89	0.49	1.92	3.86
	F	96	46.77	0.31	3.05	40.49	53.48						
L6CC	M	73	41.54	0.42	3.61	35.80	53.53	0.000	^∗∗∗^	2.03	0.57	0.91	3.14
	F	96	39.51	0.37	3.67	34.75	53.83						
L6ML	M	73	34.72	0.42	3.55	29.52	45.74	0.000	^∗∗∗^	2.13	0.50	1.14	3.12
	F	96	32.59	0.30	2.95	28.03	44.83						
L6MG	M	73	32.21	0.32	2.77	27.60	37.79	0.000	^∗∗∗^	2.21	0.43	1.36	3.05
	F	96	30.00	0.28	2.75	25.52	37.62						
L6MC	M	73	38.28	0.33	2.82	32.98	43.51	0.025	^∗^	1.07	0.47	0.14	2.00
	F	96	37.21	0.33	3.19	31.49	46.71						
L6MB	M	73	44.81	0.39	3.32	30.14	50.37	0.019	^∗^	1.15	0.48	0.19	2.10
	F	96	43.67	0.30	2.97	38.57	49.81						
L6CB	M	73	46.94	0.29	2.46	42.40	52.24	0.000	^∗∗∗^	1.75	0.47	0.81	2.69
	F	96	45.53	0.38	3.69	33.21	51.72						
L6DB	M	73	47.88	0.46	3.92	33.84	54.07	0.000	^∗∗∗^	2.34	0.59	1.18	3.51
	F	96	34.44	0.31	3.09	28.62	46.46						

NS: not significant; ^∗^*P*<0.05, ^∗∗^*P* < 0.01, and ^∗∗∗^*P* < 0.001.

**Table 3:** Upper and lower premolars' measurements including independent sample *t*-tests comparing males to females.

Abbreviation	Gender	*n*	Mean	Standard error of mean	Standard deviation	Minimum	Maximum	Significance (2-tailed)	Significance	Mean difference	Standard error difference	95% confidence interval of the difference	
												Lower	Upper
*Upper premolars*													
U5CC	M	73	40.53	0.35	2.96	35.43	47.98	0.005	^∗∗^	1.56	0.55	0.48	2.63
	F	96	38.97	0.40	3.88	32.01	50.58						
U5CL	M	73	35.92	0.40	3.43	29.70	50.04	0.000	^∗∗∗^	2.17	0.54	1.11	3.23
	F	96	33.75	0.36	3.48	25.50	40.34						
U5CG	M	73	32.07	0.34	2.93	25.91	38.19	0.005	^∗∗^	1.59	0.56	0.49	2.69
	F	96	30.48	0.41	4.03	22.86	46.14						
U5CB	M	73	46.41	0.42	3.60	37.85	53.83	0.002	^∗∗^	1.73	0.55	0.64	2.82
	F	96	44.68	0.36	3.53	37.24	51.11						
U5MC	M	73	38.78	0.34	2.94	32.49	45.35	0.000	^∗∗∗^	1.73	0.46	0.83	2.63
	F	96	37.05	0.30	2.95	31.74	43.31						
U4MC	M	73	35.74	0.38	3.21	29.66	43.44	0.003	^∗∗^	1.46	0.49	0.49	2.42
	F	96	34.28	0.25	3.10	28.94	40.86						
U4CL	M	73	30.99	0.40	3.40	25.77	38.91	0.024	^∗^	1.22	0.53	0.16	2.27
	F	96	29.78	0.35	3.47	23.81	39.89						
U4CG	M	73	27.84	0.37	3.12	20.85	34.27	0.000	^∗∗∗^	1.72	0.48	0.78	2.66
	F	96	26.12	0.31	3.02	19.54	32.87						
U4CB	M	73	41.06	0.55	4.72	14.36	49.59	0.278	NS	0.71	0.65	−0.58	1.99
	F	96	40.35	0.38	3.72	27.66	48.53						
U4MC	M	73	34.57	0.35	2.96	26.37	39.45	0.032	^∗^	1.05	0.48	0.09	2.00
	F	96	33.53	0.33	3.23	25.41	40.55						
*Lower premolars*													
L5CC	M	73	36.08	0.44	3.78	27.98	50.75	0.002	^∗∗^	1.64	0.53	0.60	2.68
	F	96	34.44	0.31	3.09	28.62	46.46						
L5CL	M	73	31.32	0.34	2.92	26.34	37.48	0.142	NS	0.74	0.50	-0.25	1.74
	F	96	30.58	0.35	3.47	25.62	45.87						
L5CG	M	73	29.74	0.37	3.16	24.87	38.55	0.606	NS	0.57	1.10	-1.61	2.75
	F	96	29.17	0.92	9.00	23.42	87.64						
L5MC	M	73	34.87	0.33	2.81	30.90	43.34	0.028	^∗^	1.00	0.45	0.11	1.90
	F	96	33.86	0.30	2.99	27.51	44.70						
L5CB	M	73	40.05	0.39	3.32	29.80	46.50	0.013	^∗^	1.37	0.54	0.29	2.44
	F	96	38.68	0.37	3.65	30.26	44.74						
L4CC	M	73	31.23	0.29	2.49	24.89	36.53	0.102	NS	0.66	0.40	-0.13	1.46
	F	96	30.57	0.27	2.67	24.82	37.16						
L4MC	M	73	27.87	0.28	2.41	23.33	33.49	0.005	^∗∗^	1.06	0.37	0.32	1.79
	F	96	26.81	0.24	2.38	21.86	31.37						
L4CG	M	73	26.55	0.36	3.05	22.28	37.35	0.012	^∗^	1.21	0.48	0.26	2.16
	F	96	25.34	0.32	3.10	19.79	35.15						
L4CB	M	73	34.02	0.39	3.34	24.08	41.57	0.629	NS	0.22	0.46	-0.69	1.14
	F	96	33.79	0.27	2.69	25.46	38.68						
L4MC	M	73	29.14	0.33	2.82	20.60	37.62	0.031	^∗^	0.77	0.35	0.07	1.47
	F	96	28.37	0.18	1.77	24.31	31.41						

NS: not significant; ^∗^*P* < 0.05, ^∗∗^*P* < 0.01, and ^∗∗∗^*P* < 0.001.

**Table 4:** Upper and lower anterior teeth measurements including independent sample *t*-tests comparing males to females.

Abbreviation	Gender	*n*	Mean	Standard error of mean	Standard deviation	Minimum	Maximum	Significance (2-tailed)	Significance	Mean difference	Standard error difference	95% confidence interval of the difference	
												Lower	Upper
*Upper anterior teeth*													
U3CL	M	73	25.95	0.29	2.44	21.79	31.23	0.147	NS	0.70	0.48	−0.25	1.65
	F	96	25.25	0.36	3.51	16.23	36.92						
U3CB	M	73	33.88	0.29	2.47	26.76	39.67	0.680	NS	0.17	0.41	−0.64	0.97
	F	96	33.71	0.28	2.74	28.92	41.78						
U3MC	M	73	27.40	0.27	2.31	22.46	34.49	0.649	NS	−0.31	0.67	−1.63	1.02
	F	96	27.70	0.55	5.35	19.94	57.25						
U2MC	M	73	16.81	0.12	1.04	14.55	19.12	0.886	NS	0.05	0.37	−0.68	0.79
	F	96	16.76	0.31	3.05	12.26	42.73						
*Lower anterior teeth*													
L3CG	M	73	21.76	0.30	2.55	17.02	31.93	0.000	^∗∗∗^	1.33	0.34	0.66	2.00
	F	96	26.73	0.26	2.19	22.46	33.15						
L3CB	M	73	26.73	0.26	2.19	22.46	33.15	0.031	^∗^	0.71	0.33	0.06	1.35
	F	96	26.02	0.21	2.03	20.96	29.94						
L3MC	M	73	21.45	0.15	1.32	18.74	25.31	0.007	^∗∗^	0.59	0.22	0.16	1.01
	F	96	20.86	0.15	1.44	16.47	22.99						
L2MC	M	73	10.81	0.11	0.95	9.18	13.11	0.090	NS	0.23	0.14	−0.04	0.50
	F	96	10.58	0.08	0.81	8.70	12.40						

NS: not significant; ^∗^*P* < 0.05, ^∗∗^*P* < 0.01, and ^∗∗∗^*P* < 0.001.

**Table 5:** Anterior palatal length (APL), palatal length (PL), total palatal length (TPL), anterior mandibular length (AML), mandibular length (ML), total mandibular length (TML), and palatal depth (PD) measurements including independent sample *t*-tests comparing males to females.

Abbreviation	Gender	*n*	Mean	Standard error of mean	Standard deviation	Minimum	Maximum	Significance (2-tailed)	Significance	Mean difference	Standard error difference	95% confidence interval of the difference	
												Lower	Upper
APL	M	73	11.10	0.96	8.16	5.95	78.60	0.134	NS	1.28	0.85	−0.40	2.97
	F	96	9.82	0.16	1.59	6.78	14.67						
PL	M	73	31.98	0.56	4.75	2.91	53.11	0.107	NS	0.94	0.58	−0.21	2.08
	F	96	31.04	0.28	2.72	22.91	38.26						
TPL	M	73	44.42	0.63	5.34	5.03	51.73	0.182	NS	0.87	0.65	−0.41	2.16
	F	96	43.55	0.31	3.04	34.31	50.12						
AML	M	73	6.27	0.13	1.14	3.52	8.75	0.276	NS	0.20	0.18	−0.16	0.56
	F	96	6.07	0.12	1.20	3.60	10.52						
ML	M	73	26.10	0.25	2.14	20.95	30.23	0.587	NS	0.22	0.41	−0.58	1.03
	F	96	25.88	0.30	2.95	20.94	48.42						
TML	M	73	40.76	0.64	5.50	4.75	52.60	0.014	^∗^	1.71	0.69	0.35	3.07
	F	96	39.05	0.35	3.44	15.31	47.82						
PD	M	73	20.90	0.24	2.08	15.02	24.21	0.264	NS	0.36	0.32	−0.28	1.00
	F	96	20.54	0.21	2.09	14.72	24.80						

NS: not significant; ^∗^*P* < 0.05.

**Table 6:** Comparison of intercanine (upper canine cusp to cusp) widths between Saudis (different studies), Caucasians, and Southern Chinese.

Population	Males				Females			
	*n*	Mean	SE	SD	*n*	Mean	SE	SD
Saudis (present study)	73	33.88	0.29	2.47	96	33.71	0.28	2.74
Saudis [22]	60	33.9	—	2.29	60	32.58	—	2.58
Saudis [21]	71	35.43	—	1.6	68	33.69	—	1
Caucasians [20]	60	34.05	—	2.1	83	32.77	—	2.2
Southern Chinese [9]	166	36.92	0.23	2.9	124	35.09	0.32	3.5

*n*, number; SE, standard error; SD, standard deviation.

**Table 7:** Comparison of intermolar (upper first molar central fossa to central fossa) widths between Saudis (different studies).

Population	Males				Females			
	*n*	Mean	SE	SD	*n*	Mean	SE	SD
Saudis (present study)	73	45.38	0.34	2.9	96	43.42	0.34	3.32
Saudis [21]	60	46.38	—	3.24	60	44.29	—	3.03

*n*, number; SE, standard error; SD, standard deviation.

**Table 8:** Comparison of intermolar (upper first molar distal buccal cusp to distal buccal cusp) widths between Saudis, Caucasians, and Southern Chinese.

Population	Males				Females			
	*n*	Mean	SE	SD	*n*	Mean	SE	SD
Saudis (present study)	73	53.25	0.33	2.8	96	51.23	0.34	3.3
Caucasians [20]	56	45.59	—	2.5	77	44.28	—	2.1
Southern Chinese [9]	209	56.17	0.19	2.8	130	54.15	0.22	2.6

*n*, number; SE, standard error; SD, standard deviation.

**Table 9:** Comparisons of maxillary arch length and depth between Saudis and Yemenis.

Population	Males				Females			
	*n*	Mean	SE	SD	*n*	Mean	SE	SD
*Anterior palatal length*								
Saudis (present study)	73	11.1	0.96	8.16	96	9.82	0.16	1.59
Yemenis [11]	113	8.88	—	1.08	101	8.54	—	1.35
*Palatal length*								
Saudis (present study)	73	31.98	0.56	4.75	96	31.04	0.28	2.72
Yemenis [11]	113	29.71	—	2.02	113	30.39	—	2.12
*Total palatal length*								
Saudis (present study)	73	44.42	0.63	5.34	96	43.55	0.31	3.04
Yemenis [11]	113	42.62	—	2.32	101	42.3	—	2.43
*Palatal depth*								
Saudis (present study)	73	20.90	0.24	2.08	96	20.54	0.21	2.09
Yemenis [11]	113	21.17	—	1.51	101	20.71	—	1.39

*n*, number; SE, standard error; SD, standard deviation.

## References

[1] Hart T. R., Cousley R. R. J., Fishman L. S., Tallents R. H. (2015). Dentoskeletal changes following mini-implant molar intrusion in anterior open bite patients. *The Angle Orthodontist*.

[2] Singh D. P., Garg A. K., Singh S. P., Krishna Nayak U. S., Gupta M. (2014). Comparison of the dental arch changes in patients with different malocclusions. *Indian Journal of Dental Research*.

[3] Ugolini A., Cerruto C., Di Vece L. (2015). Dental arch response to Haas-type rapid maxillary expansion anchored to deciduous vs permanent molars: a multicentric randomized controlled trial. *The Angle Orthodontist*.

[4] Taner T. U., Ciğer S., El H., Germeç D., Es A. (2004). Evaluation of dental arch width and form changes after orthodontic treatment and retention with a new computerized method. *American Journal of Orthodontics and Dentofacial Orthopedics*.

[5] Hua X., Xiong H., Han G., Cheng X. (2012). Correction of a dental arch-width asymmetric discrepancy with a slow maxillary contraction appliance. *American Journal of Orthodontics and Dentofacial Orthopedics*.

[6] Lundstrom A. (1949). An investigation of 202 pairs of twins regarding fundamental factors in the aetiology of malocclusion. *The Dental Record*.

[7] Meredith H. V., Cox G. C. (1954). Widths of the dental arches at the permanent first molars in children 9 years of age. *American Journal of Orthodontics*.

[8] Heithersay G. (1961). Further observations on the dentition of the Australian aborigine at Haast’s Bluff. *Australian Dental Journal*.

[9] Ling J. Y. K., Wong R. W. K. (2009). Dental arch widths of Southern Chinese. *The Angle Orthodontist*.

[10] Aluko I. A., DaCosta O. O., Isiekwe M. C. (2009). Dental arch widths in the early and late permanent dentitions of a Nigerian population. *Nigerian Dental Journal*.

[11] Al-Zubair N. M. (2015). Determinant factors of Yemeni maxillary arch dimensions. *Saudi Dental Journal*.

[12] Ward D. E., Workman J., Brown R., Richmond S. (2006). Changes in arch width: a 20-year longitudinal study of orthodontic treatment. *The Angle Orthodontist*.

[13] McWade R. A., Mamandras A. H., Hunter W. S. (1987). The effects of Fränkel II treatment on arch width and arch perimeter. *American Journal of Orthodontics and Dentofacial Orthopedics*.

[14] Mack P. J. (1981). Maxillary arch and central incisor dimensions in a Nigerian and British population sample. *Journal of Dentistry*.

[15] Abu Alhaija E. S. J., Qudeimat M. A. (2003). Occlusion and tooth/arch dimensions in the primary dentition of preschool Jordanian children. *International Journal of Paediatric Dentistry*.

[16] Tsujino K., Machida Y. (1998). A longitudinal study of the growth and development of the dental arch width from childhood to adolescence in Japanese. *The Bulletin of Tokyo Dental College*.

[17] Noroozi H., Saeeda R. (2000). Interrelationships between the width, depth, and perimeter of the dental arch. *The International Journal of Adult Orthodontics and Orthognathic Surgery*.

[18] Walkow T. M., Peck S. (2002). Dental arch width in Class II Division 2 deep-bite malocclusion. *American Journal of Orthodontics and Dentofacial Orthopedics*.

[19] Heikinheimo K., Nystrom M., Heikinheimo T., Pirttiniemi P., Pirinen S. (2012). Dental arch width, overbite, and overjet in a Finnish population with normal occlusion between the ages of 7 and 32 years. *The European Journal of Orthodontics*.

[20] Van der Linden F., Boersma H., Zelders T., Peters K. A., Raaben J. H. (1972). Three-dimensional analysis of dental casts by means of the optocom. *Journal of Dental Research*.

[21] Hashim H. A., Al-Ghamdi S. (2005). Tooth width and arch dimensions in normal and malocclusion samples: an odontometric study. *The Journal of Contemporary Dental Practice*.

[22] Abd-el Samad Younes S. (1984). Maxillary arch dimensions in Saudi and Egyptian population sample. *American Journal of Orthodontics*.

